# Tobacco consumption and premenstrual syndrome: A case-control study

**DOI:** 10.1371/journal.pone.0218794

**Published:** 2019-06-21

**Authors:** María del Mar Fernández, Agustín Montes-Martínez, María Piñeiro-Lamas, Carlos Regueira-Méndez, Bahi Takkouche

**Affiliations:** 1 Department of Preventive Medicine, University of Santiago de Compostela, Santiago de Compostela, Spain; 2 Centro de Investigación Biomédica en Red de Epidemiología y Salud Pública (CIBER-ESP), Madrid, Spain; Ordu University, TURKEY

## Abstract

**Objective:**

To assess whether tobacco smoking is associated with Premenstrual Syndrome (PMS) and its most severe form, Premenstrual Dysphoric Disorder (PMDD).

**Design:**

Case-control study with incident cases using the Spanish public healthcare system.

**Setting:**

3 major public hospitals and one family counseling and planning center.

**Population:**

Women consulting for troubles related to menstruation and for other motives such as screening for uterine cancer, contraception counseling or desire for pregnancy.

**Methods:**

Logistic regression.

**Main outcome measures:**

Odds Ratios of PMS and PMDD.

**Results:**

285 incident PMS cases and 285 age-matched controls on the one hand, and 88 incident PMDD cases and 176 controls on the other hand participated in the study. The odds of premenstrual disorders was higher in current smokers compared with never smokers: Odds Ratio (OR) = 1.78, 95% Confidence Interval (CI): 1.20–2.63 for PMS and OR = 2.92, 95%CI: 1.55–5.50 for PMDD. For PMS, women who smoke 1 to 5 cigarettes/day presented an OR = 2.82, 95%CI: 1.57–5.06 and those who smoke more than 15 cigarettes/day an OR = 2.52, 95%CI: 0.99–6.40.

Compared to non-smokers, current and ex-smokers who smoked < 3 pack-years presented an OR = 1.79, 95%CI: 1.04–3.08 for PMS, and an OR = 3.06, 95%CI: 1.27–7.35 for PMDD. Smokers of 3 to 8 pack-years presented an OR = 2.34, 95%CI: 1.33–4.13 for PMS and OR = 3.56, 95%CI: 1.55–8.17 for PMDD.

These results were confirmed by the exposure-effect curve obtained from a cubic spline model.

**Conclusions:**

This study shows that smokers are more likely to develop PMS and PMDD.

## Introduction

Premenstrual disorders are defined as the set of recurrent physical and psychic symptoms in the luteal phase of the menstrual cycle. Within these disorders, two main categories are to be highlighted: premenstrual syndrome (PMS) and premenstrual dysphoric disorder (PMDD), a more severe form [1]. Both clinical presentations have a common pathophysiological basis, although they are classified separately [2].

The prevalence sways between 20 and 40% for PMS and between 3 and 8% for PMDD in women of childbearing age in the United States [3], while a meta-analysis found that PMS affects about half of the female population worldwide, with the lowest prevalence in Switzerland (10%) and the highest in Iran (98%) [4].

The PMS symptoms affect normal daily activities, and interfere with work, studies, and interpersonal relationships [3,5]. Women who suffer from these symptoms have a 9-fold increased risk of reporting one week a month of malaise, with most of the consequences described above [3].

An American study estimated the annual costs of PMS at $ 5000 per woman per year [3]. It is remarkable that a large proportion of the cases is not diagnosed, as women do not consult a physician for the symptoms and sometimes because physicians have difficulties in establishing a diagnosis of PMS [6], or because they consider it a mere cultural and social construct and not a real disease [7]. As a consequence, the burden of women seeking medical help and receiving a diagnosis is small and is probably declining [8].

Tobacco affects the regulation of sex hormones such as estrogens, progesterone and androgens as well as the regulation of gonadotropic hormones and previous studies have linked sex hormones to the pathophysiology of PMS [9]. In particular, it was observed that the intensity of premenstrual symptoms augments proportionally to the increase in the concentrations of estrogen and progesterone in the luteal phase [10].

The World Health Organization considers tobacco consumption among women as an epidemic. Tobacco smoking has very serious consequences for the health of the woman, the fetus and the neonate as a passive smoker [11]. A report of the Surgeon General suggests that smoking can alter the menstrual function by increasing the risk of dysmenorrhea, amenorrhea and irregular menstruation [12]. All these problems cause a dramatic increase in the costs for health systems, and lead to a worse health for women and their families [11]. Furthermore, the World Health Organization calls for further studies on tobacco smoking in relation to aspects of menstruation [11]. Except for African countries in which female smoking prevalence is low, about one-fifth of women in Europe and America are current smokers. About 17% of women are smokers in the US, 24% in Spain and 20% in the UK [11].

The scarce studies that are available on the topic suggest that tobacco consumption is more common in women with PMS or PMDD [13–16]. However, it is not clear whether tobacco is a cause or a consequence of these syndromes as, with the exception of a prospective study [17], the studies are of cross-sectional nature.

The objective of our study is, therefore, to investigate to what extent tobacco smoking is related to the occurrence of premenstrual syndrome.

## Methods

### Study population

We set up a case-control study with newly diagnosed cases of PMS and PMDD. We selected 285 cases of PMS among women consulting for troubles related to menstruation and 285 controls. We also selected 88 cases of PMDD and 176 individually controls. Cases and controls were selected from 3 major public hospitals and one family counseling and planning center in the city of Santiago de Compostela, which attend a population of 400,000 users. Controls were individually age-matched to cases in each center.

The study was authorized by the regional Ethics Committee. All participants signed a consent form.

### Data collection

Data were collected through a voluntary, anonymous and self-completed questionnaire. Potential cases and controls were selected by the health personnel (gynecologist, midwife) in charge of the consultation.

To determine cases, we used the Premenstrual Syndrome Screening Tool (PSST) [18]. This questionnaire consists of 19 questions about physical, behavioral and psychological symptoms in the 5 days before the menstruation of the last three months. The severity of each symptom is graded from 1 to 4 (1: none, 4: very intense). Symptoms such as irritability and nervousness have a higher weight than physical symptoms. This, together with the higher or lower score obtained, allows to differentiate between PMS and PMDD.

To define a PMS or PMDD case, the following algorithm was used. First, for PMS, a score ≥ 3 was needed in one of the 4 questions about whether the woman felt "irritable," "tense," "tearful," or "depressed". A score of 4 in one of these questions was necessary to define a case of PMDD. In addition, to define a case of PMS, a score ≥ 3 was required in one of the 5 variables of interference with "work performance, relationship with colleagues, family members, in social life or in household tasks". A score of 4 was necessary to define a case of PMDD. The last condition was to present a score ≥ 3 in at least 4 of the first 14 questions (that is, all questions except the 5 questions related to interference) for both PMS and PMDD. Both case definitions follow the guidelines proposed in the PSST. When a patient fulfilled the case definition, we proceeded to assess exposure as explained below. Patients that did not meet the requirements were excluded from the study.

Controls were selected from women who attended the same consultation for motives other than PMS such as screening for uterine cancer, contraception counselling or desire for pregnancy. Absence of PMS was confirmed using the same questionnaire as above. In order to be included as controls, women had to present a score ≤ 2 on each of the 4 variables that describe whether they felt "irritable", "tense", "tearful" or "depressed" and also a score ≤ 2 on all variables of interference which were described earlier. After being included as a control, tobacco exposure was assessed as we did for the cases. Women who did not meet case or control definitions were excluded from the study.

The exposure questionnaire consisted of a series of items about tobacco smoking habits as well as symptoms related to menstruation, socio-demographic factors, constitutional factors, and other lifestyle factors that could possibly be confounders of the relationship between PMS and tobacco. A food frequency questionnaire was also used to determine usual intake of macro and micronutrients [19]. To assess tobacco consumption, we used the questionnaire of the World Health Organization [20]. For this purpose, we created 4 variables: 1) smoking status, with the categories "never smoker", "current smoker" and "ex-smoker", 2) quantity of pack-years of tobacco smoked by smokers and ex-smokers, 3) time since quitting for ex-smokers, and 4) number of units consumed per day in current smokers.

### Statistical analysis

We used unconditional logistic regression to obtain crude and adjusted odds ratios along with their 95% confidence intervals. This method was preferred to the conditional logistic regression to increase precision in the estimates without any loss of validity [21]. We conducted independent analyses for PMS and PMDD.

Those covariates that changed the estimate of the OR of tobacco consumption by more than 10% were introduced in the final model [22]. This model included the following variables: age, previous pregnancies, age of menarche, Body Mass Index (BMI) and educational level. The following covariates were considered for their possible inclusion in the model as confounders, but were eventually ruled out due to their lack of effect: health center, menstrual regularity, history of abortions, use of oral contraception, use of intrauterine device and antidepressant treatment. The following candidate variables were also discarded from the final model for the same reason: alcohol consumption, physical activity, intake of various nutrients (calcium, caffeine, histamine, tyramine, omega 3 and 6 fatty acids, beta-carotene, lutein, protein, total fat, saturated fatty acids, monounsaturated fatty acids, cholesterol, carbohydrates, fiber, sodium, potassium, magnesium, manganese, phosphorus, iron, copper, zinc, chlorine, selenium, iodine, vitamins, nitrates, nitrites, amino acids and total consumption of kilocalories). The analyses were performed with STATA, version 12.

To explore the shape of the curve that relates tobacco consumption to the occurrence of episodes of PMS and PMDD, we fitted a model with cubic splines adjusted for potential factors of confounding. We set 3 knots in the limits of exposure categories (0, 0.1–2.9, 3–7.9 and ≥ 8 pack-years). The number of knots as well as the type of cubic spline regression were determined using the Bayesian Information Criterion (BIC).

## Results

285 cases with PMS and 285 controls (case-control ratio 1:1), as well as 88 cases with PMDD and their corresponding 176 controls (case-control ratio 1:2) participated in this study. The response rate was 80% in cases and 80% in controls. The average age was 32 years for cases and controls, both for PMS and PMDD. Due to the low number of partial missing data, and thus, the fact that these missing data could not sensibly modify the results, we did not perform any imputation procedure. Subjects with partial missing data were not included in the analysis.

In Table 1 we observe that PMS cases had an earlier menarche and a lower body mass index than controls, as well as a higher proportion of nulliparous women. Likewise, we observe that a high proportion of the sample has a university level of education, due to the fact that the city in which this study was carried out is a university city. This proportion is higher in cases of PMS than in controls. In Table 2, we observe that the distribution of the variables among PMDD cases and their controls is very similar to that observed for PMS cases and their controls.

**Table 1:** Distribution of 285 premenstrual syndrome cases and 285 age-matched controls according to social, anthropometric and gynecological variables.

Characteristics	Category	N° cases	%	N° controls	%
Body mass index (Kg/m2)*	<19	22	8.1	14	5.2
	19–24.9	182	67.4	160	59.9
	25–30	41	15.2	62	23.2
	>30	25	9.3	31	11.6
Educational level*	Primary	46	16.3	46	16.1
	Secondary	80	28.4	111	38.9
	University	156	55.3	128	44.9
Menarche age*	< = 11	74	26.1	48	16.8
	12 y 13	153	54.1	162	56.8
	> = 14	56	19.8	75	26.3
**Number of pregnancies**	0	147	51.6	131	46.0
	1	66	23.2	70	24.6
	> 1	72	25.3	84	29.5
**Number of abortions**	0	233	81.8	229	80.4
	1	43	15.1	49	17.2
	> 1	9	3.2	7	2.5
Oral contraception*	No	181	80.8	187	81.0
	Yes	43	19.2	44	19.0
Intrauterine device*	No	235	92.9	240	91.3
	Yes	18	7.1	23	8.7

*The sum of different categories is < total number of cases or controls due to partial missing data

**Table 2:** Distribution of 88 premenstrual dysphoric disorder cases and 176 age-matched controls according to social, anthropometric and gynecological variables.

Characteristics	Category	N° cases	%	N° controls	%
Body mass index (Kg/m2)*	<19	8	9.6	9	5.4
	19–24.9	56	67.5	108	65.1
	25–30	14	16.9	35	21.1
	>30	5	6.0	14	8.4
Educational level*	Primary	18	20.9	31	17.6
	Secondary	23	26.7	72	40.9
	University	45	52.3	73	41.5
**Menarche age**	< = 11	21	23.9	27	15.3
	12 y 13	50	56.8	98	55.7
	> = 14	17	19.3	51	29.0
**Number of pregnancies**	0	47	53.4	74	42.0
	1	18	20.5	48	27.3
	> 1	23	26.1	54	30.7
**Number of abortions**	0	69	78.4	147	83.5
	1	13	14.8	24	13.6
	> 1	6	6.8	5	2.8
Oral contraception*	no	57	80.3	112	80.0
	yes	14	19.7	28	20.0
Intrauterine device*	no	72	93.5	147	93.0
	yes	5	6.5	11	7.0

*The sum of different categories is < total number of cases or controls due to partial missing data

### Effect of tobacco consumption on PMS

The association of PMDD with tobacco is shown in Table 3. Current tobacco consumption was associated with increased occurrence of PMS (OR = 1.78, 95% CI: 1.20–2.63). Ex-smokers are also twice more likely than non-smokers to present PMS, although the confidence interval of the association is wider than for current smoking due to the small sample size of this exposure group (OR = 1.98, 95% CI: 0.92–4.23).

**Table 3:** Crude and adjusted odds ratios (OR) of tobacco consumption and premenstrual syndrome.

Characteristics	Category	N° cases	%	N° controls	%	Crude OR	95% CI	Adjusted OR*	95% CI
**Smoking status**	**never**	138	51.3	171	62.2	1.00		1.00	
	**current**	111	41.3	88	32.0	1.56	1.09–2.24	1.78	1.20–2.63
	**ex-smoker**	20	7.4	16	5.8	1.66	0.82–3.40	1.98	0.92–4.23
**Cigarettes / day**	**0**	138	56.6	171	67.1	1.00		1.00	
	**1–5**	51	20.9	22	8.6	2.87	1.66–4.97	2.82	1.57–5.06
	**6–15**	41	16.8	53	20.8	0.96	0.60–1.52	1.12	0.68–1.85
	**> 15**	14	5.7	9	3.5	1.93	0.81–4.59	2.52	0.99–6.40
**Pack-years**	**Never smoker**	138	52.7	171	63.6	1.00		1.00	
	**0.1–2.9**	43	16.4	35	13.0	1.54	0.93–2.55	1.79	1.04–3.08
	**3.0–7.9**	48	18.3	27	10.0	2.21	1.31–3.72	2.34	1.33–4.13
	**≥ 8.0**	33	12.6	36	13.4	1.11	0.65–1.03	1.29	0.73–2.30
**Time without smoking (years)**	**Never smoker**	138	87.3	171	91.9	1.00		1.00	
	**1–3**	5	3.2	6	3.2	1.02	0.31–3.43	1.14	0.33–3.96
	**3.1–8**	7	4.4	4	2.2	2.12	0.60–7.43	2.35	0.56–9.92
	**> 8**	8	5.1	5	2.7	1.90	0.60–6.07	2.24	0.69–7.33

* Adjusted for age, previous pregnancies, menarche age, Body Mass Index and educational level.

**Fig 1:**
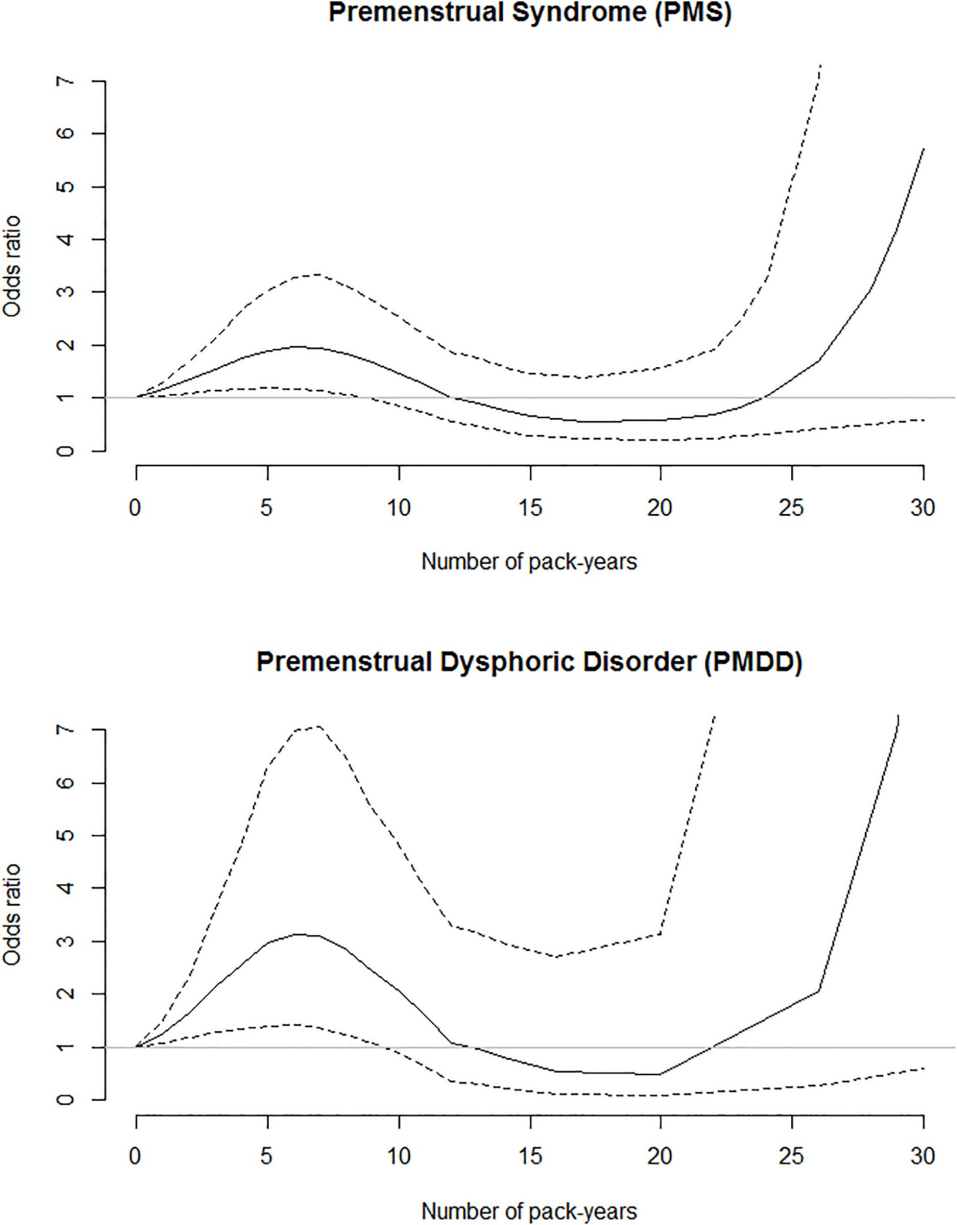
Adjusted odds ratios of PMS and PMDD, calculated by cubic spline regression according to pack-years of tobacco from a case-control study. Continuous line: point estimates; dotted lines: 95% confidence intervals.

Compared to non-smokers, current and ex-smokers whose total quantity of pack-years consumed was less than 3 pack-years were more likely to develop PMS (OR = 1.79, 95% CI: 1.04–3.08). For those who smoked 3 to 8 pack-years the OR was 2.34 (95%CI: 1.33–4.13). The spline curve (Fig 1) shows an increase in the odds of PMS for a tobacco consumption of less than 10 pack-years. The odds decreases for a consumption of 10 to 25 pack-years but shows an exponential increase for higher consumptions, albeit with imprecise estimates due to the small sample size, as shown by the amplitude of the confidence interval.

As for daily consumption, women who smoke between 1 and 5 cigarettes/day and those who smoke more than 15 cigarettes/day are more likely to suffer from PMS than non-smokers (OR = 2.82, 95%CI: 1.57–5.06 and OR = 2.52, 95% CI: 0.99–6.40, respectively).

Due to the low number of ex-smokers in our sample, the analysis of the time elapsed since quitting their addiction in relation to PMS yielded results with confidence intervals that were too wide to draw reliable conclusions.

### Effect of tobacco consumption on PMDD

The association of PMDD with tobacco is shown in Table 4. As in PMS, current smoking is related to PMDD (OR = 2.92, 95% CI: 1.55–5.50). Furthermore, women who had smoked less than 3 pack-years (OR = 3.06, 95%CI: 1.27–7.35) and those who had smoked between 3 and 8 pack-years (OR = 3.56, 95%CI: 1.55–8.17) were also more likely to develop PMDD than nonsmokers. As in the analysis of PMS, the effect observed in smokers of 8 and more pack-years is lower and its confidence interval is imprecise.

**Table 4:** Crude and adjusted odds ratios (OR) of tobacco consumption and premenstrual dysphoric disorder.

Characteristics	Category	N° cases	%	N° controls	%	Crude OR	95% CI	Adjusted OR*	95% CI
**Smoking status**	**never**	37	43	106	62.4	1.00		1.00	
	**current**	43	50.0	54	*31*.*8*	2.28	1.31–3.98	2.92	1.55–5.50
	**ex-smoker**	6	7.0	10	*5*.*9*	1.73	0.58–5.20	1.70	0.52–5.57
**Cigarettes / day**	**0**	37	16.8	106	67.5	1.00		1.00	
	**1–5**	19	24.1	12	7.6	4.56	2.02–10.31	4.53	1.82–11.24
	**6–15**	20	25.3	35	22.3	1.63	0.84–3.17	2.19	1.03–4.65
	**> 15**	3	3.8	4	2.5	2.18	0.46–10.21	3.87	0.70–21.34
**Pack-years**	**Never smoker**	37	44.1	106	63.9	1.00		1.00	
	**0.1–2.9**	17	20.2	18	*10*.*8*	2.78	1.28–6.05	3.06	1.27–7.35
	**3.0–7.9**	20	23.8	20	*12*.*1*	2.86	1.39–5.90	3.56	1.55–8.17
	**≥ 8.0**	10	11.9	22	*13*.*3*	1.25	0.52–2.97	1.62	0.64–4.12
**Time without smoking (years)**	**Never smoker**	37	86.0	106	91.4	1.00		1.00	
	**1–3**	1	2.3	4	3.4	0.72	0.78–6.71	0.67	0.57–7.87
	**3.1–8**	3	7.0	3	2.6	2.60	0.49–13.76	1.59	0.26–9.79
	**>8**	2	4.7	3	2.6	1.58	2.35–10.58	2.11	0.29–15.39

* Adjusted for age, previous pregnancies, menarche age, Body Mass Index and educational level.

The spline curve that relates exposure to tobacco to occurrence of PMDD (Fig 1) presents a very similar form to that relating tobacco and PMS, with a significant increase in the odds for low consumption and imprecise estimates for higher consumptions.

As for the number of cigarettes consumed per day, we observed that light and moderate smokers are related to the occurrence of PMDD. Heavy smokers present a relation of similar magnitude, albeit with a wider confidence interval.

The diagnosis of PMS and PMDD is based essentially on the evaluation of variables related to mood. These variables could overlap with factors that are related to psychological stress. In order to rule out any effect due to stress, we carried out a second analysis restricted to those participants who presented a low level of perceived stress (156 subjects for PMS analysis and 81 subjects for PMDD analysis), measured by the scale proposed by Cohen et al. [23]. After restriction, the results still showed a strong relation of tobacco smoking with PMS and PMDD: OR for current smokers 3.21 (95% CI: 1.28–8.03) for PMS and 8.55 (95% CI: 1.55–47.03) for PMDD.

## Discussion

Our results suggest that women who smoke are more likely to suffer from both PMS and PMDD. The fact that they have ever smoked increases the odds of suffering both forms of the syndrome. Current smoking is clearly associated with increased PMS/PMDD. However, although the effect persists in women who quit smoking, the association observed is imprecise due to the reduced number of ex-smokers in our study.

Our results are in accordance with the only prospective study carried out so far on the relation between smoking and the development of PMS [17]. The magnitude of the effect is similar in both studies. However, that study showed a dose-response effect of tobacco and PMS, something our study did not reveal, possibly due to differences in consumption categories between the two studies. Furthermore, our results are also consistent with results of other cross-sectional studies, although it should be highlighted that these are more prone to reverse causality bias that might explain their findings [13–15, 24, 25].

It has been shown that tobacco decreases the activity of monoamine oxidase (MAO), an enzyme that degrades serotonin, a neurotransmitter involved in the pathophysiology of PMS [26]. This inhibition seems to be related to the amount of tobacco and the time of consumption [26]. During smoking, MAO is strongly inhibited, but its synthesis is increased considerably in order to compensate for this inhibition [26]. It is then plausible that quitting from smoking may increase serotonin degradation. This could then explain the increasing probability of developing PMS and PMDD as abstinence time augments.

Previous reports favor the hypothesis of a relationship between dysmenorrhea and PMS [27]. It is likely that estrogens exert an effect on both PMS and dysmenorrhea [10, 28]. In our study, additional adjustment for the presence of dysmenorrhea decreased the magnitude of the relation between tobacco and PMS (data not shown). In view of the association of PMS and dysmenorrhea, it is probable that dysmenorrhea could play the role of an intermediate variable in the causal path between tobacco and PMS. Adjusting for the variable “dysmenorrhea” would then cause an overadjustment and, therefore, would introduce additional bias in the assessment of the relation between tobacco and PMS [29].

As in any case-control study, our study may be subject to recall bias. In theory, PMS cases may better remember their tobacco intake than controls. However, this is unlikely to occur, as, in general, smoking is a stable habit throughout life [30]. Also, different studies have manifested that self-reporting of tobacco consumption is a valid method for measuring tobacco exposure when compared to biological measures such as measurement of cotinine or carbon monoxide [31]. In addition, our diagnosis of PMS was not openly revealed to the participants, since it consisted of a score that originated from several independent questions. The hypothesis of the study, i.e. the relation between tobacco intake and premenstrual syndrome, was not disclosed to the participants, since tobacco smoking was only one among several exposure factors that have been assessed in the exposure questionnaire.

We cannot rule out a certain amount of misclassification in the PMS/PMDD assessment since the diagnosis is based on a subjective symptom score. However, this misclassification bias, if any, is unlikely to modify the conclusion of this study, first because the screening tool used to diagnose PMS was previously validated; although not in our population but in a similar one [32]. Second, because if there is any erroneous classification, it is likely that this error occurs regardless of the exposure status, since the subjects were not aware of the hypothesis of the study that related smoking and PMS.

To explore the direction and magnitude of the potential bias due to misclassification of outcome, we reanalyzed the data by introducing the variable "smoking" as dichotomous, first as ever smoker/never smoker and then as current smoker/current non-smoker. The resulting odds ratios were for PMS 1.56, 95% CI: 1.11–2.20) and 1.49, 95% CI: 1.05–2.12) respectively, and for PMDD 2.19, 95% CI: 1.29–3.72) and 2.15, 95% CI: 1.26–3.66). These results, suggest that the true association between smoking and PMS may even be stronger than the one we have observed, as non-differential misclassification of a dichotomous variable yield bias towards the null value, that is, decreases the effect [33].

Some of our findings are limited by the small sample size in some categories of exposure. This had lead to estimates with wide confidence intervals. Also, generalizability of the findings may be a concern due to the fact that the study was carried out among women with high education level.

## Conclusion

In this study we observed that smoking is related to both PMS and PMDD. Future research should determine how long it takes to eliminate the effect of tobacco on PMS and PMDD after quitting. Also, it would be of interest to determine whether smoking may have an *in utero* effect, increasing the risk of PMS in women whose mothers smoked during pregnancy. As for any etiologic research, cross-sectional designs are best avoided.

The World Health Organization expects an increase in tobacco consumption among women in emerging countries, which are the most densely populated and those which harbor the highest birth rates [11]. Tobacco harm is personal, environmental and transgenerational. Given the very large number of women currently concerned by the syndrome and in anticipation of future affectation, it is important to decide whether health authorities should warn against the risk that tobacco consumption induces in the development of PMS / PMDD.

## Supporting information

S1 File Questionnaire Original Spanish version. (PDF)

S2 File Questionnaire English translation. (DOCX)

S3 File Database PMS. (DTA)

S4 File Database PMDD. (DTA)

S5 File Variables definition. (ODT)
